# Modulation of Leukemic Blasts into Dendritic Cells (DC_leu_) and Their Role in Predicting Survival in Patients with AML and MDS

**DOI:** 10.3390/cancers18050847

**Published:** 2026-03-06

**Authors:** Daniel Christoph Amberger, Zuzana Fischer, Diana Deen, Anika Hirn-Lopez, Caroline Plett, Alexander Rabe, Christoph Schwepcke, Selda Ugur, Lara Kristina Klauer, Christian Ansprenger, Anja Liepert, Markus Freudenreich, Christoph Schmid, Helga Maria Schmetzer

**Affiliations:** 1 Department of Medicine III, University Hospital of Ludwig-Maximilian-University Munich, 81377 Munich, Germany; 2 First Department of Medicine, Paracelsus Medical University, 5020 Salzburg, Austria; 3 Bavarian Cancer Research Center (BZKF), 80539 Munich, Germany; 4 Department of Haematology and Oncology, University Hospital of Augsburg, 86156 Augsburg, Germany

**Keywords:** AML, leukaemia-derived dendritic cells, DC/DC_leu_-generating protocols, anti-leukaemic activity

## Abstract

Background/Objectives: Acute myeloid leukemia (AML) is characterized by impaired anti-leukemic immune responses, and the ex vivo or in vivo generation of dendritic cells (DCs), including leukemic dendritic cells (DC_leu_), represents a promising strategy to stimulate immune cells and improve anti-leukemic activity. Methods: This study examined the generation, phenotype and functional relevance of DCs and DC_leu_ produced ex vivo from blast-containing PBMNCs and whole blood (WB) in AML. Using both standard DC/DC_leu_-generating protocols and available Kits. Results: We show that DC/DC_leu_ can be reliably generated with both methods. Generated DC/DC_leu_ effectively activated T cells during mixed lymphocyte cultures (MLCs), resulting in enhanced anti-leukemic cytotoxicity. Improved blast lysis correlated with specific immunological features, including higher frequencies of generated DC_leu_ and mature DC subsets, as well as a certain cytokine pattern after DC/DC_leu_ cultures or MLC. In addition, the frequencies of proliferating T cells after MLC strongly correlated with the degree of achieved blast lysis, underscoring the importance of efficient DC/DC_leu_-mediated T cell stimulation. Both the frequencies of generated DC/DC_leu_ and the resulting blast lytic activity were linked to overall survival (OS) in AML patients. Individuals who failed to demonstrate improved blast lysis exhibited significantly reduced OS, suggesting inadequate immune responsiveness of patients in vivo. Conclusions: These findings identify phenotypic and functional immune parameters as predictors of clinical outcome and highlight the prognostic relevance of ex vivo immune profiling. This approach may help to optimize and personalize future immunotherapeutic strategies in AML.

## Simple Summary

Ex vivo-generated dendritic cells (DC/DC_leu_) from leukemic whole blood or isolated PBMNCs effectively activate immune effector cells and enhance anti-leukemic cytotoxicity. Higher frequencies of DC/DC_leu_ and distinct cytokine secretion patterns are associated with improved blast lysis and improved immune responses in vivo. These functional immune parameters correlate with overall survival of patients, underscoring the prognostic relevance of generated DC/DC_leu_. Thus, monitoring DC/DC_leu_ generation and immune activation may provide valuable guidance for individualized immunotherapeutic strategies and improved treatment stratification in AML patients.

## 1. Introduction

Acute myeloid leukemia (AML) is a clonal disorder of genetically altered hematopoietic stem cells, marked by uncontrolled proliferation of immature myeloid blast cells, impairing normal haematopoiesis [1,2].

The diagnosis of AML is based on cellular, cytogenetic and molecular characterization of leukemic cells in the peripheral blood and bone marrow [3]. Induction chemotherapy, using an anthracycline–cytarabine combination followed by consolidation chemotherapy, remains the standard treatment for fit patients with AML [4]. In recent years, novel agents such as Midostaurin (a multitarget tyrosine kinase inhibitor for AML with FLT3 mutations) and Venetoclax (a B-cell lymphoma-2 inhibitor) for elderly or multi-comorbid AML patients unfit for intensive chemotherapy have been incorporated into treatment regimens, improving clinical response [5,6,7]. Hematopoietic stem cell transplantation (HSCT), based on the graft-versus-leukemia effect, remains a potentially curative post-remission therapy for AML [8,9,10]. The prognosis in AML largely depends on chromosomal abnormalities and clinical factors such as age, performance status and previous cytotoxic treatments. Unfortunately, the prognosis remains poor, with a 5-year overall survival rate of 30%, highlighting the need for durable treatment options [11,12].

Advancements in the molecular pathophysiology of AML have led to the development of various heterogeneous immunotherapies. These include antibody-based therapies targeting antigens expressed on leukemic (stem) cells (e.g., CD33, CLEC12A), immunoconjugates, bispecific T-cell engagers (BiTEs), dual-affinity retargeting antibodies (DARTs) and immune checkpoint inhibitors [13,14,15,16,17]. Additionally, adoptive immunotherapy has emerged, including genetically engineered T cells expressing chimeric antigen receptors (CARs) and T-cell receptor (TCR)-modified T cells, which are designed to reactivate effector cells against leukemic blasts [18,19]. Moreover, strategies targeting specific antigens, such as Wilms tumor 1 (WT1), have also been developed [20,21].

Dendritic cells (DCs), as major antigen-presenting cells (APCs), are a promising target for adjuvant treatment in relapsed/refractory AML or to stabilize remissions [22]. Potentially, two main sources of DCs are used or could be used in AML immunotherapy clinical trials: CD14^+^ monocyte-derived DCs (mo-DCs) and leukemia-derived DCs (DC_leu_). Mo-DCs can be generated ex vivo under Good Manufacturing Practice (GMP) conditions from peripheral blood mononuclear cells (PBMNCs) or isolated monocytes and loaded with defined leukemia-associated antigens (LAAs) using electroporation of messenger RNA (mRNA) or by peptide pulsing [23,24]. Several strategies are known to produce (mature) DC_leu_ from PBMNCs. After pulsing or ex vivo generation, those DC/DC_leu_ can be adoptively administered as a vaccine [25,26,27,28]. Alternatively, DC/DC_leu_ can be generated from (blast-containing) leukemic whole blood (WB) without induction of blast proliferation ex vivo in the presence of immunomodulators using standard DC/DC_leu_-generating protocols (e.g., MCM, Ca, PICI, INTRON), as listed in Table 1 [29]. WB-based DC/DC_leu_ generation models most closely simulate physiological conditions and the tumor microenvironment of AML patients, as they include all patient-specific cellular and soluble components and thus closely reflect the physiological (potentially inhibitory or activating) tumor microenvironment in AML. We have developed ten different Kits (Kit A, C, D, E, F, G, H, I, K, M), which combine two or three clinically approved immune-modulatory compounds and enable the generation of mature DC_leu_ directly from leukemic blasts [30,31] (Table 1).

PBMNC- and DC/DC_leu_-generating protocols/Kits combine cytokines such as granulocyte-macrophage colony-stimulating factor (GM-CSF), Interleukin 4 (IL-4) or FLT3 ligand (FL) for the induction of myeloid differentiation; danger signals (e.g., Picibanil or nucleic acids); and maturation/danger signaling factors such as tumor necrosis factor-α (TNF-α), prostaglandin E1 (PGE_1_) or prostaglandin E2 (PGE_2_) to promote DC/DC_leu_ differentiation and maturation. Importantly, these combinations support differentiation without triggering blast proliferation [33].

DC/DC_leu_ produced with DC/DC_leu_-generating protocols or Kits have been shown to activate (adaptive and innate) immune cells after T cell-enriched immunoreactive cells in Mixed Lymphocyte Cultures (MLC): they promote T cell proliferation and induce effective anti-leukemic responses [33]. The activation of immune cells is monitored through flow cytometry, tracking up/downregulation of different cell subtypes, leukemic blasts, and chemokines [33]. To optimize our WB model, we ranked the Kits based on their ability to generate DC/DC_leu_ and initiate anti-leukemic cytotoxicity in T cell-enriched immunoreactive cells. The top-performing Kits — Kit I (GM-CSF + Picibanil), Kit K (GM-CSF + PGE_2_) and Kit M (GM-CSF + PGE_1_)—were selected as the most promising combinations of clinically approved immune-response modifiers [31]. Correlation analyses showed positive correlations between the quantity of DC/DC_leu_ generated with Kit I and Kit M (vs. controls) and induced anti-leukemic effects after MLC [33]. These (ex vivo) anti-leukemic effects induced after MLC of Kit M-pretreated WB were independent of patients’ response to induction chemotherapy, disease stage, French–American–British (FAB) classification, European LeukemiaNet (ELN) risk stratification, platelet/white blood/red blood cell counts, age and sex [34].

These data obtained with DC/DC_leu_-generating protocols show that those cells could be prepared and adoptively transferred to patients. Moreover, a Kit-based immunotherapy (treating patients directly with Kits) could lead in vivo to induced DC/DC_leu_, followed by DC/DC_leu_-activated immune cells that could contribute directly to induced/sustained remissions in AML patients, independent of clinical prognostic and diagnostic criteria.

In this study, we further investigated the quantity and quality of DC/DC_leu_ generated from both AML-blast-containing PBMNCs and WB and assessed their ability to activate immunoreactive cells after T cell-enriched MLC, correlating the frequencies of DC/DC_leu_ (generated from leukemic WB with various DC/DC_leu_-generating protocols or Kits) with cytokines released into culture supernatants and with induced anti-leukemic activity. We also correlated various clinical parameters (e.g., stage of disease, response to induction therapy) with the quality and quantity of DC/DC_leu_ subtypes to predict AML patients’ overall survival (OS) and to contribute to identifying Kits with potential to improve anti-leukemic responses in vivo.

## 2. Material and Methods

### 2.1. Sample Collection and Preparation

Samples were obtained from randomly selected patients with AML (*n* = 76) or high-grade myelodysplastic syndrome (*n* = 3) in active stages of the disease after written informed consent was obtained in accordance with the local Ethics Committee (Pettenkoferstraße 8a, 80336 Munich, Ludwig Maximilians University Hospital; Vote No. 19-034). Samples were provided by the University Hospitals of Tuebingen, Düsseldorf, Munich, Oldenburg, and Augsburg. Anticoagulation was performed using lithium-heparin tubes (7.5 mL, Sarstedt, Nürnberg, Germany) containing standardized concentrations of heparin. PBMNCs were isolated from blood samples by density gradient centrifugation using Ficoll-Hypaque (density of separating solution 1.077 g/mL, Biochrom KG, Berlin, Germany). T cells were positively selected from PBMNCs via magnetic beads using CD3^+^ microbeads and column-based immunomagnetic cell separation technology (Miltenyi Biotec, Bergisch Gladbach, Germany). Cell counts were determined using Neubauer counting chambers. PBMNCs and T cells were frozen until use at −80 °C using Dimethysulfoxide or in liquid nitrogen according to standardized procedures.

### 2.2. Patients’ Characteristics and Definitions

Blood samples were collected from patients with AML (with an average age of 52.3 years, range 5–85 years and a female-to-male ratio of 1:1) and classified according to the French–American–British classification (FAB) (M0–M7), aetiology of AML (primary, secondary) and stage of the disease (diagnosis = dgn., persistence = pers., relapse = rel. and relapse after HSCT = rel.a.HSCT) [35]. AML patients at diagnosis were classified as favorable (*n* = 5), intermediate (*n* = 15) or adverse (*n* = 13) risk groups according to ELN criteria [4]. AML patients’ response to therapy was determined by achieving or not achieving complete remission (defined as <5% blasts in bone marrow and no blasts in peripheral blood 30 days after therapy start). An overview of patients’ characteristics is given in Table 2.

The cellular composition of blood samples used for subsequent experiments was 59 ± 31% leukemic blasts, 4 ± 18% CD19^+^ B cells, 11 ± 7% CD3^+^ T cells, 2 ± 13 CD56^+^ NK cells and 4 ± 23% CD14^+^ monocytes as detected by Fluorescence-activated Cell Sorting (FACS). In cases with aberrant expression of T, B, NK or monocytoid antigens on blasts, frequencies were not included in the analysis.

### 2.3. Flow Cytometry

Flow cytometry using a panel of mouse monoclonal antibodies (mAbs) directly conjugated with fluorescein isothiocyanate (FITC) (^a^), phycoerythrin (PE) (^b^), tandem Cy7-PE (PC7) (^c^) or allophycocyanin (APC) (^d^) was used to analyse frequencies and subtypes of cells before and after cultures. Antibodies were obtained from Becton Dickinson (Heidelberg, Germany) (CD1a^b^, CD1b^a^, CD14^c^, CD15^d^, CD71^c^, CD206^d^, 7AAD^c^, CCR7^c^), Beckman Coulter (Krefeld, Germany) (CD1^ab^, CD3^a^, CD19^c^, CD33^d^, CD56^b^, CD80^b^, CD117^b,d^, CD206^b^, CD34^a,c^, CD65^a^, CD83^a^) and Thermo Fisher Scientific (Darmstadt, Germany) (CD1a^b^, CD13^c^, CD34^d^ and CD86^a^).

For the flow cytometric analyses, samples were suspended in PBS with 5–20% fetal calf serum (FCS, Biochrom, Berlin, Germany), incubated with mAbs for 15 min, washed, centrifuged and resuspended in 100–200 µL PBS. WB samples were additionally treated with lysing buffer (Becton Dickinson, Heidelberg, Germany) according to manufacturer’s instructions. Isotype controls were used according to manufacturer’s instructions.

Flow cytometric analyses were performed with FACS Calibur Flow Cytometer (Becton Dickinson, Heidelberg, Germany) and analysed with CellQuest (Software version 5.2.1) data acquisition and analysis software (Becton Dickinson, Heidelberg, Germany).

Frequencies of mature DC/DC_leu_ subtypes and T cells were quantified by flow cytometry [29]. An overview of quantified cells is given in Table 3.

### 2.4. Cell Culture Experiments

All cell culture experiments, including DC/DC_leu_ cultures, MLCs and a cytotoxicity fluorolysis assay, were set up under standard laboratory conditions (37 °C, 21% O_2_ and 5% CO_2_).

### 2.5. Dendritic Cell Culture from PBMNCs and WB

DC/DC_leu_ were generated from 3–4 × 10^6^ isolated leukemic PBMNCs with the DC/DC_leu_-generating protocols MCM-Mimic (MCM), Calcium ionophore (Ca), Picibanil (Pici) and Interferon (INTRON) [29,32]. Cells were pipetted into 12-multiwell tissue culture plates (ThermoFisher Scientific, Darmstadt, Germany) and diluted in 2 mL serum-free X-Vivo-15 medium (Lonza, Basel, Switzerland). Cytokines were added, as described in Table 1. Half-medium exchange was carried out after 3–4 cell culture days. A culture without added response modifiers served as a control.

DC/DC_leu_ were generated from leukemic WB (presenting the physiological cellular and soluble composition of the individual samples) with Kits A, C, D, E, F, I, K and M [31]. Then, 500 μL of WB was pipetted in 12-multiwell-plates and diluted 1:2 in X-Vivo-15 medium (Lonza, Basel, Switzerland) to imitate physiological conditions. Response modifiers and immune-modulating factors were added to cultures as described in Table 1. A culture without added response modifiers served as a control.

### 2.6. Mixed Lymphocyte Cultures (MLC)

First, 1 × 10^6^ thawed patients’ CD3^+^ T cells were co-cultured with IL-2 and a stimulator cell suspension containing approximately 2.5 × 10^5^ generated DC/DC_leu_, which were generated with the DC/DC_leu_-generating protocol or Kits from leukemic PBMNCs or WB (MLC^PBMNC DC^; MLC^WB DC^). MLCs of T cell-enriched immunoreactive cells with a stimulator cell suspension without pretreatment with DC/DC_leu_-generating protocol or Kits (MLC^PBMNC^; MLC^WB^) served as a control, as previously described [36]. Next, the cells were harvested after 6–7 days, and the subtypes were quantified by flow cytometry and subsequently used for a cytotoxicity fluorolysis assay.

### 2.7. Cytotoxicity Fluorolysis Assay

The blast lytic activity of DC/DC_leu_-stimulated T cell-enriched immunoreactive cells in MLC from PBMNC and WB cultures (containing effector cells) was analyzed in a Cytotoxicity Fluorolysis Assay (CTX), as previously described [31,34].

The lytic activity of effector cells was defined as the percentual difference between the frequencies of viable blast target cells before and after T effector cell contact (achieved lysis). To assess the lytic activity of effector cells in more detail, we defined the percentual difference between the achieved lysis of standard DC/DC_leu_ protocol/Kit-pretreated PBMNC/WB vs. untreated PBMNC/WB in every given case (improved lysis).

### 2.8. Enzyme-Linked Immunosorbent Assay (ELISA)

Secretion of Interleukin 10 (IL-10), Interleukin 17 (IL-17) and Monocyte Chemoattractant Protein-1 (MCP-1) was analyzed in cell culture supernatants (stored at −80°C until the day of analysis) collected from DC/DC_leu_ cultures generated from WB (IL-10DC, IL-17DC, MCP-1DC) as well as from MLCs (IL-10MLC, IL-17MLC, MCP-1MLC) using Sandwich ELISA immunoassay Kits (R&D Systems, Abingdon, UK). Detection limits of the sandwich ELISA immunoassay Kits were set by the manufacturer (R&D Systems) as follows: IL-10: 11 pg/mL; IL-17: 0.5 pg/mL; and MCP-1: 15 pg/mL. The samples were evaluated with a Tristar LB941 ELISA reader (Berthold company, Bad Wildbach, Germany) using a standard curve according to the manufacturer’s instructions.

### 2.9. Statistical Methods

We evaluated descriptive statistics (average, median, standard deviation) and conducted paired and unpaired *t*-tests, Mann–Whitney U test, Chi-squared Test, Pearson correlation coefficients and Kaplan–Meier analyses. Statistical analysis and figures were implemented with SPSS Statistics 28 (IBM Software, New York, NY, USA) and GraphPad Prism 8 (GraphPad Software, CA, USA). Differences were considered ‘not significant’ (n.s.) with *p* values > 0.10, as ‘borderline significant’ with *p* values from 0.10 to 0.05, and as ‘significant’ with *p* values < 0.05. Correlation was defined as ‘no correlation’ in cases with r values between 0 and 0.1, as ‘low’ in cases with r values between 0.2 and 0.4, as ‘medium’ in cases with r values between 0.5 and 0.7, and as ‘high’ in cases with r values > 0.7.

## 3. Results

It is well known that DC/DC_leu_ can be generated from leukemic blasts in PBMNCs as well as WB, which present the physical, cellular and soluble components of each individual patient. These generated DC/DC_leu_ can specifically activate immune cells against leukemic blasts after MLC, resulting in effective anti-leukemic activity against leukemic blasts.

**1.** 
**Blasts are regularly converted to DC/DC_leu_ in the presence of standard DC/DC_leu_-generating protocols and Kits, leading to activated ne cells, after stimulation in T cell-enriched MLC**

Pooling all data obtained with standard DC/DC_leu_-generating protocols from PBMNCs (compared to MCM alone) and all data with Kits from WB (compared to Kit I alone), comparable frequencies of DC_leu_/cells, DC_leu_/bla^+^, DC_leu_/DC^+^ and DC_mat_/DC^+^ were found after DC/DC_leu_ cultures. Frequencies of generated DC_leu_ and subsets increased significantly using DC/DC_leu_-generating protocols and Kits compared to control without added response modifiers (Supplementary Materials Figure S1(A1,A2)), thereby confirming data previously shown [29,31]. No increase in proliferating blasts was found before vs. after Kit treatment. Analysis of T cell subsets before and after T cell-enriched MLC with DC/DC_leu_-containing PBMNCs or WB showed consistent results, confirming a shift toward higher T cell activation. This effect was independent of whether DC/DC_leu_ were generated using standard protocols from PBMNCs or with Kits from WB.

Furthermore, we analyzed the concentrations of IL-10, IL-17 and MCP-1 in DC/DC_leu_ culture supernatants after cultures with Kits (pooling all data with all Kits compared to Kit I pretreated samples alone) (Supplementary Materials Figure S1(B1,C1,D1)) or from supernatants collected after MLC (pooling all data with all Kit-pretreated samples (compared to Kit I pretreated samples alone)) (Supplementary Materials Figure S1(B2,C2,D2)). We found no differences in cytokine levels when pooling all data from WB cultures pretreated with Kits vs. Kit I alone. However, we found higher levels of all cytokines compared to control supernatants without added Kits, as has been previously described [36].

In summary, our data support previously published findings that DC/DC_leu_ subtypes can be reliably generated from blast-containing PBMNCs using standard protocols, as well as from whole blood using Kits. Additionally, these cells are capable of stimulating T cells to a more activated state during MLC and to an increased release of chemokines and cytokines to DC/DC_leu_ or MLC culture supernatants.

**2.** 
**Functional analyses**

We evaluated the potential of DC/DC_leu_ to induce and improve T cells’ anti-leukemic activity after MLC: We analysed blast lytic activity of T cells stimulated with generated DC/DC_leu_ from PBMNCs and WB (MLC^DCPBMNC^ and MLC^DCWB^) as well as their respective controls (T cells stimulated with leukemic PBMNCs and WB without previous stimulation with generated DC/DC_leu_ (MLC^PBMNC^ and MLC^WB^)) in a cytotoxicity assay. The lytic activity of effector cells (MLC^DCPBMNC^, MLC^DCWB^) versus their respective controls (MLC^PBMNC^, MLC^WB^) was analyzed after different coculture durations with blast target cells: at 3 h, at 24 h and additionally by selecting the ‘best lytic result per case’ from either the 3 or 24 h time points in cases where both measurements were available.

  **2.1**  **Higher probability of achieving blast lysis after DC/DC_leu_ stimulated T cell enriched MLC**

First, we examined the anti-leukemic potential of DC/DC_leu_-stimulated effector cells after MLC to lyse blasts. We found more cases with achieved blast lysis after T cell stimulation with DC/DC_leu_ (MLC^DCPBMNC^) vs. control (MLC^PBMNC^) after MLC with DC/DC_leu_ generated with standard DC/DC_leu_ methods (Supplementary Materials Figure S2(A1)). In all conditions, effector cells lysed more blasts than in the control group (Supplementary Materials Figure S2(A2)). These stimulations enhanced blast lysis across all DC/DC_leu_-generating methods (Supplementary Materials Figure S2(B1)), with up to a 100% increase in blast lysis observed after MCM pretreatment (Supplementary Materials Figure S2(B2)) compared to controls.

Pooling all data on effector cell cytotoxicity after MLC with DC/DC_leu_ generated using **Kits** (MLC^DCWB^) compared to MLC with blast-containing WB (MLC^WB^), we also found in all cohorts more cases with achieved blast lysis after T cell stimulation with DC/DC_leu_ (MLC^DCWB^)-containing cells (Supplementary Materials Figure S2(C1))**.** In all settings, more blasts were lysed compared to control (Supplementary Materials Figure S2(C2)). These stimulations resulted in up to 100% of cases compared to control, depending on the Kits used (Supplementary Materials Figure S2(D1)).

In summary, stimulation of T cells with generated DC/DC_leu_ from PBMNC and WB leads to an increase in blast lysis compared to control.

**3.** 
**DC/DC_leu_, blast, T cell and cytokine profiles in correlation with anti-leukemic activity after MLC**

To evaluate the predictive role of DC/DC_leu_, T cell and cytokine values and their subtypes for the mediation or improvement of anti-leukemic reactions (vs. controls), we correlated frequencies/concentrations of cellular/soluble compositions of the samples with their anti-leukemic potential. In the first step, we present pooled results obtained with all standard DC/DC_leu_-generating methods/Kits; in the second step, we present results obtained with single standard DC/DC_leu_-generating methods/Kits: The role of cellular/soluble composition on achieved blast lysis (vs. no lysis) or improved (vs. not improved) blast lysis as compared to controls was evaluated.

Pooling all data obtained with standard DC/DC_leu_-generating methods and analysing DC/DC_leu_ subsets after culture of PBMNCs with standard DC/DC_leu_-generating methods showed increased frequencies of DC^+^/cells, DC_leu_/cells, DC_leu_/bla^+^ and DC_mat_/DC^+^ in samples with achieved (vs. not achieved) lysis after 3 h or 24 h, as well as when selecting the best result after 3 or 24 h (e.g., %DC_leu_/bla^+^: 32 ± 25 vs. 24 ± 15). When analysing T cell populations after MLC with DC/DC_leu_ generated with standard DC/DC_leu_ methods, a significantly increased frequency of T_prol_ was found in samples with achieved (vs. not achieved) lysis when selecting the best result after 3 or 24 h (%T_prol_/CD3^+^: 42 ± 40 vs. 11 ± 9%, *p* = 0.005). When analysing data obtained with single standard DC/DC_leu_ methods, MCM (*n* = 44), Ca (*n* = 16) and PICI (*n* = 16) yielded comparable results. All in all, we confirmed previous data that (mature) DC/DC_leu_ subtypes are increased under the influence of standard DC/DC_leu_-generating methods leading to activated T cells and improved blast lysis.

Pooling all data obtained with Kits (*n* = 20) and analysing DC/DC_leu_ subsets after culture of WB with Kits showed increased frequencies of DC_leu_/DC^+^ in samples with achieved (vs. not achieved) lysis after 3 h or 24 h, as well as when selecting the best result after 3 or 24 h. Increased frequencies were found for %DC_leu_/bla^+^ (37 ± 23 vs. 27 ± 15%, *p* = 0.029). When analysing T cell populations, significantly increased frequencies of CD8^+^ T cells were found after MLC with DC/DC_leu_ generated with Kits in samples with achieved (vs. not achieved) lysis (e.g., %T_CD8_^+^/CD3^+^: 51 ± 16 vs. 40 ± 18, *p* = 0.079).

Analysing cytokine release in DC/DC_leu_ culture supernatants showed increased concentrations of IL-10 (Ø of IL-10_DC_: 0.77 ± 2.15 vs. 0.0 ± 0.0 pg/mL), decreased concentrations of MCP-1 (Ø of MCP-1DC: 679 ± 580 vs. 1212 ± 41 pg/mL) and comparable concentrations of IL-17 in supernatants of samples with achieved (vs. not achieved) lysis after 3 h, as well as when selecting the best result after 3 or 24 h of co-incubation of target with effector cells. Analysing cytokine release in MLC supernatants showed higher concentrations of IL-10 (Ø of IL-10MLC: 332 ± 441 vs. 203 ± 145 pg/mL), lower concentrations of IL-17 and MCP (Ø of IL-17MLC: 106 ± 81 vs. 166 ± 46 pg/mL; Ø of MCP-1MLC: 434 ± 493 vs. 911 ± 494) in supernatants of samples with achieved (vs. not achieved) lysis after 3 h, as well as when selecting the best result after 3 or 24 h of co-incubation of target with effector cells.

**Figure 1:**
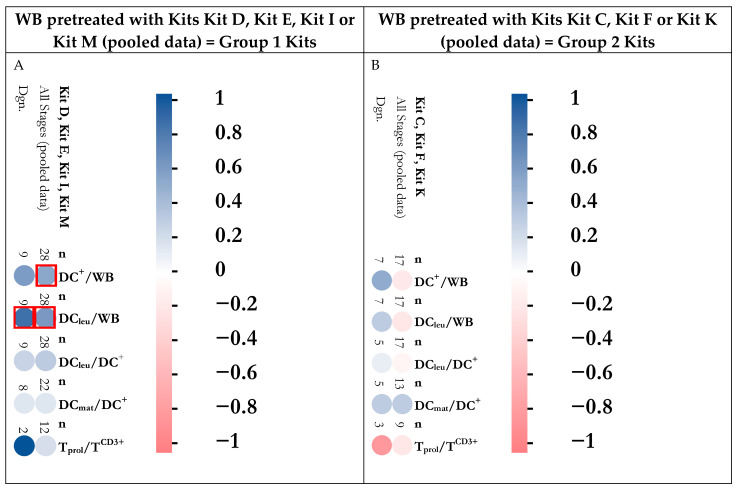
Correlation analyses (pair-wise Pearson correlation) of best achieved blast cytotoxic activity (as measured by Cytotoxicity Assay after T cell-enriched MLC with Kit-pretreated WB) with DC/DC_leu_ and T cell subtypes. Frequencies of DC/DC_leu_ and T cell subtypes in correlation with cases with superior blast lysis (group 1 Kits, samples pretreated with Kit D, Kit E, Kit I or Kit M (pooled data)) shown in (**A**) or with cases with inferior blast lysis (group 2 Kits (samples pretreated with Kit C, Kit F or Kit K (pooled data))) schown in (**B**) in AML patients at diagnosis, persistence or relapse (‘All Stages’) and at diagnosis (‘Dgn.’) are given. Pair-wise Pearson correlation was used.

To correlate achieved anti-leukemic effects with DC/DC_leu_ and T cell subsets, we grouped cases according to ‘best achieved anti-leukemic activity’ after Kit pretreatment of patients’ WB. Only cases were included in which DC/DC_leu_ subsets were available with these Kits and where cytotoxicity results after 3 h and 24 h of co-incubation of target with effector cells were available. We separated cases into two groups, characterized by ‘best achieved lysis’ of cells after T cell-enriched MLC with Kit-pretreated WB (group 1 Kits: Kit pretreatment with D, E, I, M (pooled results), Figure 1A, left side), compared to cases with inferior anti-leukemic efficacy (group 2 Kits: Kit pretreatment with Kits C, F, K (pooled results), Figure 1B, right side).

Correlation analyses showed that superior anti-leukemic activity of group 1 Kits correlated with higher frequencies of DC/DC_leu_ subtypes (after Kit culture) and proliferating T cells after MLC compared to group 2 Kits characterized by inferior achieved blast lysis; this pooled cohort correlated with lower frequencies of DC/DC_leu_ subtypes and proliferating T cells after MLC (Figure 1(A2), right side).

**4.** 
**Frequencies of generated DC/DC_leu_ and resulting blast lytic activity as predictors for response to induction therapy and for overall survival in AML patients**

**Figure 2:**
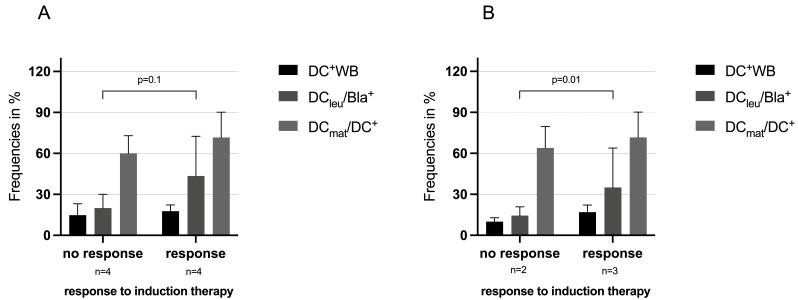
DC/DC_leu_ subtypes in AML patients according to their response to induction therapy in corresponding WB samples pretreated with group 1 Kits (Kit D, Kit E, Kit I or Kit M (pooled data, (**A**)) and with single Kit I (**B**). The average frequencies of generated DC/DC_leu_ subsets using group 1 Kits (including Kit D, Kit E, Kit I and Kit M) are presented in relation to response or non-response to induction therapy. (**A**) shows pooled data for all group 1 Kits, while (**B**) displays data for Kit I alone. Response to induction therapy was defined as <5% blasts in bone marrow and no blasts in peripheral blood 30 days after therapy start.

As shown in Figure 2 (left side), we could demonstrate that frequencies of DC/DC_leu_ subtypes (especially of %DC_leu_/Bla^+^ and DC_mat_/DC^+^, generated with group 1 Kits (Kit D, E, I or M, pooled data)) were higher in patients at first diagnosis who had responded vs. not responded to induction therapy. These differences were even more pronounced when only selecting for patients in whom DC/DC_leu_ were generated with Kit I (Figure 2, right side).

**Figure 3:**
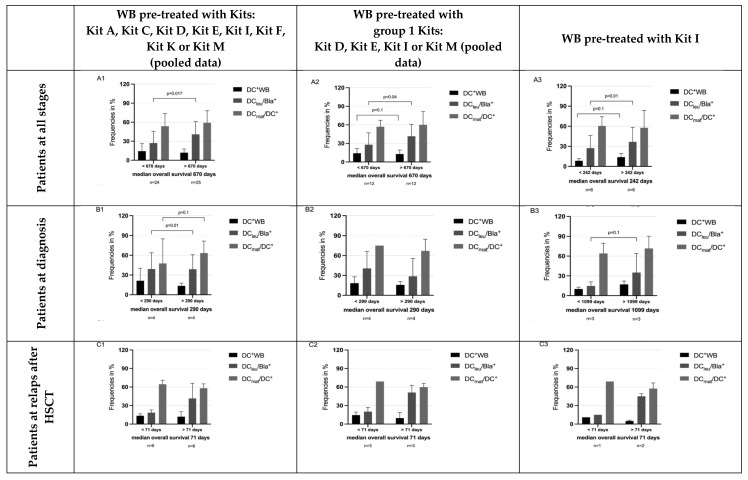
Frequencies of DC/DC_leu_ subtypes in patients with long compared to short overall survival (OS). Significantly higher frequencies of DC/DC_leu_ subtypes could be generated in patients with longer overall survival (OS) compared to patients with shorter OS than the median survival of the cohort. Figure 3 shows comparisons of generated DC/DC_leu_ subsets with OS of patients at all stages (first row, **A1**, **A2**, **A3**), only samples studied at first diagnosis (second row, **B1**, **B2**, **B3**) or at relapse after HSCT (last row, **C1**, **C2**, **C3**). Comparable results were seen, including all pooled Kits (first column, **A1**, **B1**, **C2**), only samples cultured with group 1 Kits (second column, **A2**, **B2**, **C2**) or only samples cultured in the presence of Kit I (last column, **A3**, **B3**, **C3**). We present frequencies of DC/DC_leu_ subtypes as mean ± SD in patients with OS longer (compared to shorter) than the median OS of the cohort.

Moreover, we correlated the long-term vs. short-term survival of patients (defined as survival longer or shorter than the median survival of the cohort) and found that, in all comparisons, patients with longer vs. shorter survival were characterized by significantly higher frequencies of DC_leu_/Bla^+^ in all stages of the disease, at diagnosis and at relapse after HSCT, using all Kits pooled, group 1 Kits and Kit I alone (Figure 3). In summary, this indicates that longer median survival is associated with higher generated DC_leu_ counts.

**Figure 4:**
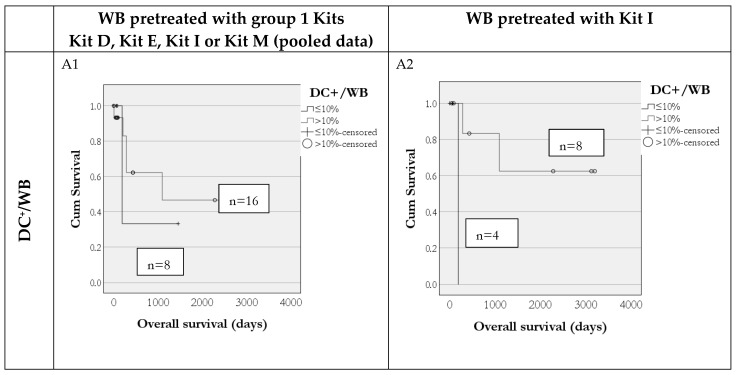

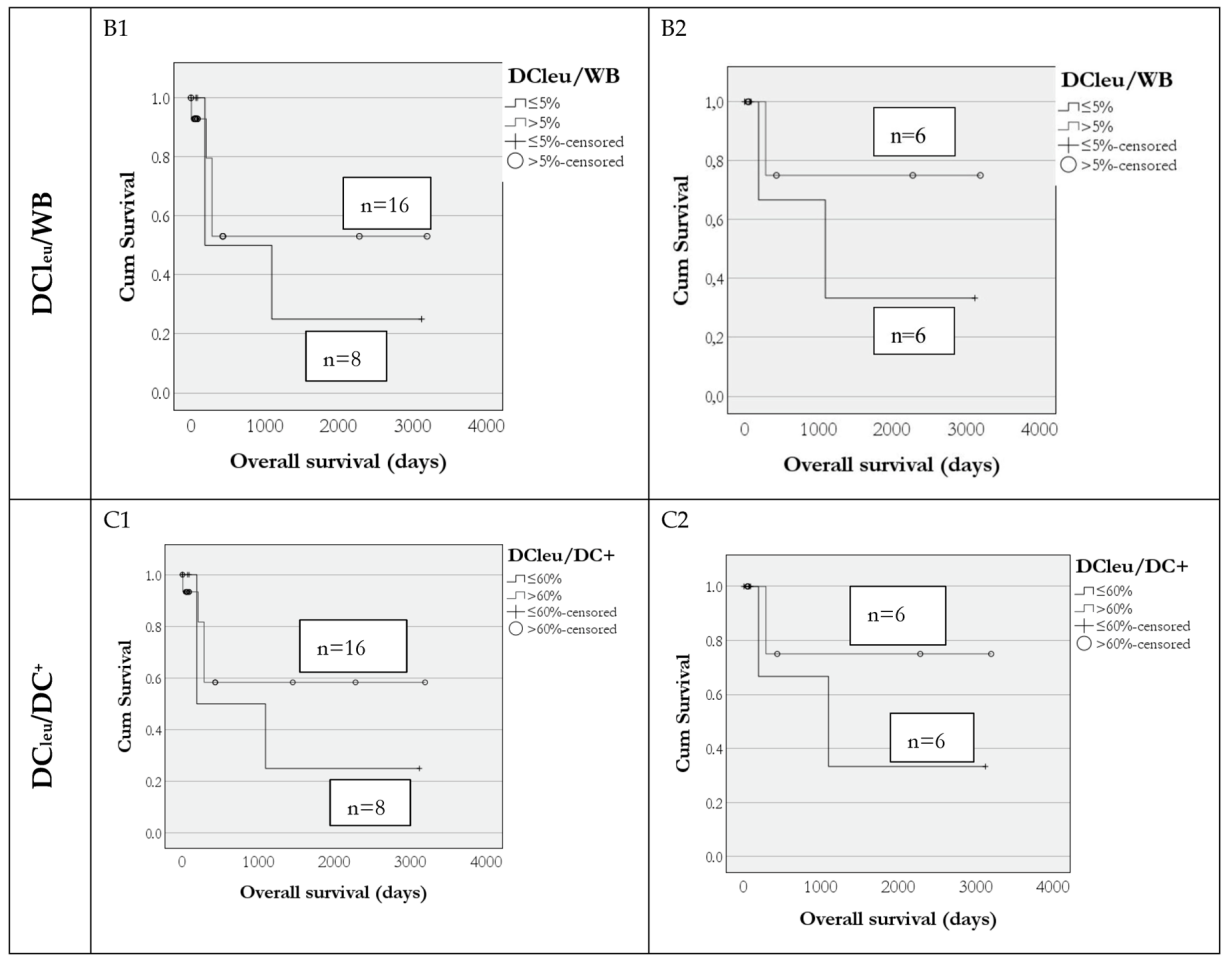
Predictivity of DC/DC_leu_ subtype frequencies for patients’ overall survival (OS). AML patients in whom higher frequencies of DC^+^/WB, DC_leu_/WB and DC_leu_/DC^+^ could be generated ex vivo were characterized by a (not significant) higher probability of overall survival. We present pooled data for group 1 Kits (including Kit D, Kit E, Kit I or Kit M) (**A1**,**B1**,**C1**) and for single Kit I (**A2**,**B2**,**C2**).

**Figure 5:**
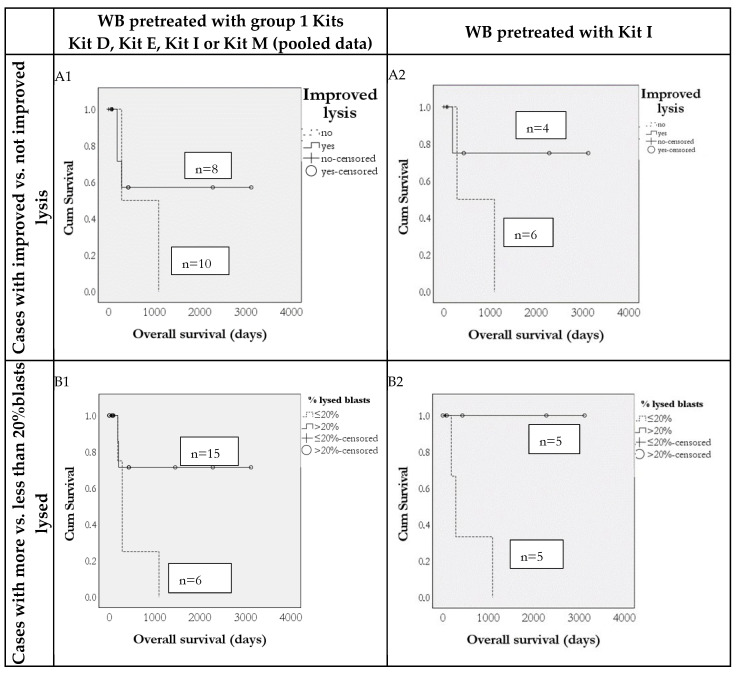
Predictivity of ex vivo anti-leukemic activity of immunoreactive cells after 3 or 24 h of stimulation with AML-blast-containing WB pretreated with Kits for patients’ overall survival (OS). AML patients in whom lysis was improved vs. untreated control were characterized by a higher probability of overall survival(OS) using pooled data for group 1 Kits (including Kit D, Kit E, Kit I and Kit M) (**A1**) and for Kit I (**A2**). AML patients in whom more than 20% of blasts were lysed were characterized by a higher probability of overall survival using group 1 Kits (including Kit D, Kit E, Kit I and Kit M) (**B1**) and for Kit I (**B2**).

Furthermore, we could show that frequencies of ex vivo-generated DC and DC_leu_, including their subgroups, are predictive for overall survival (OS) in 24 AML patients: Survival rates were higher in patients where more than 10% DC^+^/WB and more than 5% DC_leu_/DC^+^ could be generated with group 1 Kits (Kits D, E, I or M in DC/DC_leu_ cultures (data pooled)) (Figure 4,f left side). These findings were consistent both when pooling data from all Kits and when analyzing data from Kit I alone (Figure 4, right side), but not for patients whose blood was treated with group 2 kits (Kits C, F, K). Additionally, as shown in Figure 5, increased blast lytic activity was also associated with improved overall survival. In cases with non-improved blast lysis after MLC, the overall survival was reduced compared to cases with improved blast lysis.

## 4. Discussion

This study provides a comprehensive analysis of the generation and functional relevance of DC/DC_leu_ derived from blast-containing PBMNCs and WB using both established laboratory DC/DC_leu_-generating protocols and available Kits. The DC/DC_leu_-generating Kits used in this study are distinct combinations of clinically approved immune-modulatory compounds that directly influence DC/DC_leu_ differentiation, maturation, and functional capacity. Defining the relevance of each Kit allows interpretation of differences in DC/DC_leu_ quantity, quality, and induced anti-leukemic activity, defining protocols with the greatest translational potential for in vivo application.

### 4.1. Generation of DC/DC_leu_ and T Cell Activation

Our results confirm that leukemic blasts can consistently be differentiated into functionally competent DC/DC_leu_ using both standard DC/DC_leu_-generating protocols and Kits [29,31,37] (Supplementary Materials Figure S1). These generated cells exhibited robust expression of key dendritic cell markers and were capable of inducing T cell activation during MLC. Importantly, this activation translated into enhanced anti-leukemic responses in vitro, supporting earlier studies and further validating the immunogenic potential of DC/DC_leu_ [34]. Similar efficacy across PBMNCs and WB underscores their flexibility and practical relevance, particularly for clinical settings where sample availability may vary.

### 4.2. Cytokine Modulation by Kit Pretreatment

Cytokines in AML—both pro-inflammatory (e.g., IL-1β, IL-6, TNF-α, IL-17) and anti-inflammatory (e.g., IL-10)—regulate leukemic cell growth, survival, immune evasion, and chemoresistance, shaping disease progression and patient outcomes [38]. We could show that treating WB with Kits did not noticeably change the levels of major pro-inflammatory cytokines like IL-17. However, it did cause a small but consistent rise in IL-10 and MCP-1 (Supplementary Materials Figure S1). This suggests that the Kits might have mild immune-modifying effects—potentially contributing to attracting immune cells (through MCP-1) [39,40] or to promoting regulatory responses (through IL-10) [41]. While the variability in MCP-1 release was not statistically significant, it points to compositional differences between Kits that may influence the cytokine milieu and T cell dynamics. Although IL-10 is classically defined as an immunosuppressive cytokine, elevated IL-10 levels in samples with effective blast lysis may reflect strong preceding immune activation rather than a direct inhibitory effect on cytotoxicity. This observation may be explained by the fact that effector T cells, including Th1 cells, can produce IL-10 as part of an intrinsic negative feedback mechanism to limit excessive inflammation. In addition, IL-10 can exert context-dependent immunostimulatory effects. In tumor models, IL-10 has been shown to enhance CD8^+^ T-cell infiltration, increase IFN-γ expression, and support cytotoxic function, thereby promoting effective immune surveillance under specific inflammatory conditions [42,43,44]. Furthermore, lower MCP-1 levels in samples with effective blast lysis may reflect reduced recruitment of immunosuppressive monocytes and myeloid cells, promoting a microenvironment more permissive for T-cell-mediated cytotoxicity [45,46].

### 4.3. Improved Anti-Leukemic Activity Following DC/DC_leu_ Stimulation

Functionally, T cells stimulated with DC/DC_leu_ demonstrated superior anti-leukemic activity compared to those co-cultured with leukemic blasts alone. This enhanced cytotoxicity was consistently observed across both 3 h and 24 h co-culture durations. Here we could confirm previously published data from our working group [29,30,34] (Supplementary Materials Figure S2). The different results after 3 or 24 h of co-culture might be the result of different killing mechanisms or, at least, a variation of this process (e.g., the slow pathway of Fas/FasL- or the fast pathway of perforin–granzyme-mediated killing). These effects might act synergistically or independently [47,48].

### 4.4. Correlation Analyses

Our results demonstrate a clear correlation between the cellular and soluble composition of generated DC/DC_leu_, T cell activation, cytokine profiles and the anti-leukemic activity of T cells after MLC. When pooling data from all standard DC/DC_leu_-generating methods, increased frequencies of mature DC/DC_leu_ subtypes (e.g., DC_leu_/bla^+^, DC_mat_/DC^+^) were consistently associated with effective blast lysis, indicating that the quality and maturation state of generated DC/DC_leu_ are key determinants of T cell-mediated cytotoxicity [34]. The expression of CCR7 on mature DCs is crucial for the migratory capacity of DCs and DC_leu_ to the lymph node, where they activate T cells and other immunoreactive cells and induce anti-leukemic activity [49,50,51]. Correspondingly, MLC analyses showed significantly higher frequencies of proliferating T cells in samples with achieved lysis, supporting the role of DC/DC_leu_-induced T cell activation in mediating anti-leukemic effects. Single-method analyses (MCM, Ca, PICI) confirmed these observations, highlighting the robustness of standard DC/DC_leu_ generation in promoting functional immune responses [26].

When evaluating Kit-based DC/DC_leu_ generation, similar trends were observed. Samples with superior anti-leukemic activity displayed higher frequencies of DC_leu_/DC^+^ and CD8^+^ T cells, although statistical significance was marginal for some comparisons. Cytokine profiling revealed that increased IL-10 and IFN-γ concentrations in DC/DC_leu_ cultures, together with reduced MCP-1 levels, were associated with effective blast lysis, suggesting that a cytokine milieu combining immune activation (IFN-γ) and regulatory balance (IL-10) may optimize T cell function while limiting excessive inflammation [52]. In MLC supernatants, higher IL-10 and lower IL-17 and MCP-1 levels further supported a regulatory environment conducive to sustained T cell-mediated cytotoxicity [53].

Grouping cases by best achieved anti-leukemic activity after Kit pretreatment revealed that superior cytotoxic responses correlated with higher frequencies of DC/DC_leu_ subsets and proliferating T cells, whereas inferior responses were associated with lower frequencies of these key populations (Figure 1 and Figure 2). These findings collectively underscore that both the cellular composition (mature DC/DC_leu_, proliferating T cells) and the cytokine environment (IL-10, IFN-γ, MCP-1, IL-17) critically influence the efficacy of anti-leukemic T cell responses. The data support the predictive value of DC/DC_leu_ and T cell profiling for assessing and potentially enhancing the anti-leukemic capacity of immunotherapeutic strategies [25].

### 4.5. Frequencies of Generated DC/DC_leu_ and Achieved Blast Lytic Activity Can Predict Overall Survival

Our data demonstrate that longer vs. shorter survival compared to the median survival of patients studied in various stages of the disease is associated with significantly increased DC_leu_ values (Figure 3) and, vice versa, that cases with increased DC/DC_leu_ generation exhibited improved survival outcomes (Figure 4 and Figure 5). These results were consistent across pooled data from multiple Kits as well as in the subset of patients treated with Kit I alone, indicating the robustness and reproducibility of this immune signature.

In addition to phenotypic markers, functional immune activity—specifically, the ex vivo blast lytic capacity—was also predictive of clinical outcomes. Increased blast lysis in MLC following stimulation with Kit-pretreated samples was associated with better overall survival. Conversely, in cases where blast lysis was not improved after MLC, overall survival was markedly reduced. This suggests that a lack of functional anti-leukemic immune response reflects an impaired immune system, which may compromise the patient’s ability to control disease progression or respond to immunotherapeutic interventions. Kits may become valuable tools for treating patients with AML in the future, as they have the potential to enhance anti-leukemic activity in vivo. This approach could reduce the need for expensive and complicated vaccinations with ex vivo generated and manipulated (leukemic antigen-loaded) mo-DC and may ultimately improve the prognosis and relapse-free survival of patients with AML.

## 5. Conclusions

This study demonstrates that both the cellular composition and cytokine environment of generated DC/DC_leu_ critically shape T cell activation and anti-leukemic ex vivo activity. Mature DC/DC_leu_ subsets, together with proliferating and cytotoxic T cells and a certain cytokine milieu, consistently correlated with effective blast lysis with standard DC/DC_leu_-generating protocols and Kits. Importantly, higher DC/DC_leu_ frequencies and stronger ex vivo anti-leukemic cytotoxicity were associated with improved overall survival for the patients, underscoring their value as predictive biomarkers. All in all, this study highlights DC/DC_leu_-based immune profiling as a promising tool to refine and enhance immunotherapeutic strategies in AML. The results support further exploration of DC/DC_leu_ as components of personalized immunotherapy and emphasize the importance of immune competence in therapeutic response. As a next step to translate ex vivo findings from bench to bedside, the efficacy and safety of the Kit treatment have to be evaluated in appropriate preclinical in vivo models, such as AML animal models, to confirm immunogenicity and anti-leukemic activity under physiological conditions. We have already treated leukemic rats (compared to rats without treatment) as well as three patients with refractory AML with Kit M. The treatment was very well tolerated. We observed immune activation with increased leukemia-specific responses and memory cell formation. In addition, leukemic blast counts were stabilized and, in some cases, even reduced [54,55]. We are currently performing studies in leukemic mice using a different mouse model, which contributes to a clinical trial. Based on these results, prospective clinical trials would be required to assess safety, feasibility, and clinical benefit in vivo.

## Figures and Tables

**Table 1:** DC/DC_leu_-generating protocols and Kits used for the generation of DC/DC_leu_ from leukemic PBMNCs and WB.

Standard DC/DC_leu_-Generating Protocols Used for the Generation of DC/DC_leu_ from PBMNCs				
	Composition	Mode of Action	Culture Time (d)	References
MCM Mimic (MCM)	GM-CSF IL-4 FL IL-6 IL-1ß PGE_2_	Cytokine-driven DC/DC_leu_ differentiation; PGE_2_ increases the frequencies of matured DC/DC_leu_ (expressing CCR7^+^)	10–14	[29]
Calcium ionophore (Ca)	A23187	DC/DC_leu_ differentiation bypassing the standard DC/DC_leu_ generation; A23187 induces maturation	3–4	[29]
Picibanil (Pici)	GM-CSF IL-4 Picibanil (OK-432) PGE2	Cytokine-driven DC/DC_leu_ differentiation including ‘danger signaling’: lysis products from Streptococcus pyogenes and PGE_2_ stimulate DC/DC_leu_ differentiation and maturation	9–11	[29,32]
Interferon (INTRON)	GM-CSF Picibanil (OK-432) PGE_2_ TNF-α IFN-α	Cytokine-driven DC/DC_leu_ differentiation	10–12	[29]
**Kits used for the generation of DC/DC_leu_ from WB**				
Kit A	GM-CSF TNF-α		7–10	[31]
Kit C	GM-CSF PGE_2_		7–10	[31]
Kit D	GM-CSF Picibanil (OK-432) PGE_2_		7–10	[31]
Kit E	GM-CSF IFN-α		7–10	[31]
Kit F	GM-CSF A23187		7–10	[31]
Kit I	GM-CSF Picibanil (OK-432)		7–10	[31]
Kit K	GM-CSF PGE_2_		7–10	[30,31]
Kit M	GM-CSF PGE_1_		7–10	[30,31]

DC, dendritic cells; DC_leu_, leukemic-derived dendritic cells; PBMNCs, peripheral mononuclear cells; WB, whole blood; GM-CSF, granulocyte-macrophage colony-stimulating factor; IL-4, Interleukin 4; FL, FLT3 ligand; IL-6, Interleukin 6; IL-1ß, Interleukin 1 beta; PGE_1_/PGE_2_, prostaglandin E1/E2; TNF-α, tumor necrosis factor alpha; IFN-α, interferon alpha.

**Table 2:** Patient characteristics.

Pat.	Age at Dgn./Sex	FAB Type	Blast Phenotype (CD)	IC-Blasts, %	ELN-Risk	T Cell Source	Response to Therapy
**Patients at First Diagnosis (** ***n*** ** = 32)**							
407	72/m	AML M5	65, 13, 33	70	Int	Autologous	NR
436	67/f	AML M4	34, 56, 33, 13, 15, 65	45	Int	Autologous	NR
518	55/m	AML M2	34, 117, 13	86	Int	Autologous	R
546	39/m	AML M2	34, 117, 65, 15	50	Fav	Autologous	R
561	43/m	AML M2	117, 65, 15	65	Adv	Autologous	R
618	65/m	MDS RAEB II	117, 33	35	Adv	Autologous	R
748	66/m	AML M2	34, 117, 33	32	Int	Autologous	NR
757	63/f	AML M4	34, 117, 33	46	Adv	Autologous	R
820	48/m	AML-M2	34, 117, 33, 13	53	Int	Autologous	R
824	70/m	AML M1/M2	34, 7, 13	68	Adv	Autologous	R
837	69/m	AML M2	34, 117, 33, 13	39	Int	Autologous	R
898	47/f	AML M1	117, 33	87	Int	Autologous	R
948	42/f	AML M5	15, 65, 4, 33	34	Adv	Autologous	R
1144	32/m	AML M1	34, 117, 56, 33, 13, 19	82	Fav	Autologous	R
1194	72/m	n.d.	34, 117, 33	30	Adv	Autologous	R
1196	39/f	sMDS RAEB	117, 33	8	Int	Autologous	R
1201	60/f	AML-M5	15, 64, 65, 13, 14, 4, 56	41	Fav	Autologous	R
1226	69/m	n.d.	117, 34, 33	65	Adv	Autologous	NR
1245	48/f	AML M5	15, 56, 65, 33	12	Adv	Autologous	R
1251	82/m	AML M4/5	64, 13, 33, 65, 56, 4	67	n.d.	Autologous	NR
1255	58/m	AML M4	34, 56, 117, 33	15	Int	Autologous	R
1280	27/m	AML M0	34, 117, 33	88	Adv	Autologous	R
1285	56/f	AML M4	34, 117, 33, 13, 65	40	n.d.	Autologous	R
1292	44/f	AML M2	34, 117, 33, 13	70	Fav	Autologous	R
1300	24/f	AML M1	34, 117, 13, 33, 65	64	Int	Autologous	R
1306	42/m	MDS RAEB2	34, 117, 33	11	Fav	Autologous	NR
1331	45/f	AML M1	34, 13, 33, 4, 7	60	Int	Autologous	R
1335	76/f	n.d.	34, 117, 33	86	Int	Autologous	NR
1345	85/m	AML M1	117, 33, 13	96	Int	Autologous	n.d.
1350	75/m	n.d.	34, 33, 117	17	Adv	Autologous	NR
1353	70/m	AML M2	34, 117, 33, 13	63	Adv	Autologous	NR
1356	26/m	AML/ALL	34, 19, 65	80	Adv	Autologous	NR
**Patients with persisting disease (** ***n*** ** = 3)**							
502	41/m	AML M4eo	34, 117, 65, 33, 13, 15	66	Adv	Autologous	R
1024	39/m	AML M2	34, 117, 33	30	Int	Autologous	R
1165	59/m	n.d.	34, 117, 33, 13	46	Int	Autologous	R
**Patients at relapse (** ***n*** ** = 19)**							
458	50/f	AML M4	34, 38, 117, 33, 14, 64	38		Autologous	R
481	66/f	AML M4–M5	34, 117, 33, 13, 15, 56, 19	93		Autologous	R
545	44/f	AML M1	117, 34, 33	87		Autologous	NR
584	51/f	AML M1	117, 13, 33	91		Autologous	R
652	66/m	AML M1	34, 33, 13	88		Autologous	NR
655	65/f	AML M2	34, 117, 33	20		Autologous	R
914	69/m	AML M4	34, 33, 13, 15, 64, 14	43		Autologous	NR
984	63/m	AML M2	34, 117, 13, 33	42		Autologous	NR
998	67/m	AML M5	117, 34, 64, 4	91		Autologous	R
1011	57/f	AML M1	117, 34, 33, 4	61		Autologous	R
1017	69/m	AML M4	34, 117, 33	44		Autologous	R
1206	73/f	AML M2	117, 33, 15, 64	42		Autologous	NR
1231	46/f	AML M1	117, 56, 33	55		Autologous	R
1243	35/m	AML M2	34, 117, 33, 13, 2	33		Autologous	R
1303	64/f	AML M4	56, 33, 14, 65, 4	79		Autologous	R
1330	73/f	AML M4	34, 33	92		Autologous	NR
1376	53/f	AML M2	34, 117, 33	61		Autologous	NR
1386	58/m	n.d.	34, 117, 65, 33, 13	80		Autologous	NR
1388	40/f	AML M3	34/117, 33, 13	47		Autologous	NR
**Patients atrelapse after HSCT (** ***n*** ** = 25)**							
419	68/f	AML M1	34, 117, 33, 15	94		After HSCT	R
428	59/f	AML M4	34, 117, 33	35		After HSCT	NR
453	59/f	AML M1	34, 117, 33, 13	81		After HSCT	NR
460	36/m	AML M0	56, 13, 33, 65, 15, 7	60		After HSCT	NR
466	61/f	AML M6	34, 117, 65, 33, 13	52		After HSCT	NR
478	25/f	AML M0	34, 117, 56, 33, 13	33		After HSCT	R
483	33/m	AML M1	34, 117, 65, 33, 15	88		After HSCT	NR
538	12/m	AML M0	34, 65, 33	90		After HSCT	NR
565	68/f	AML M5	34, 65, 33, 13, 15	29		After HSCT	NR
610	5/f	AML M1	34, 117, 4, 33	80		After HSCT	NR
763	39/f	AML M0	34, 117, 65, 33	52		After HSCT	R
767	58/f	AML M1	34, 117, 65, 33, 13	95		After HSCT	NR
818	40/f	AML M0	34, 117, 65, 33	91		After HSCT	NR
853	34/m	AML M0	34, 117, 33, 71	89		After HSCT	NR
880	27/m	AML M4	34, 117, 33, 15	95		After HSCT	R
938	59/f	AML M4	34, 117, 33, 15	34		After HSCT	NR
980	40/f	AML M0	34, 33, 13	82		After HSCT	R
1001	60/f	AML M4	34, 117, 33	35		After HSCT	NR
1143	60/f	n.d.	117, 34, 3, 19	24		After HSCT	NR
1222	41/m	AML M1	33, 34, 117, 56	60		After HSCT	NR
1286	27/m	AML M5	34, 117, 33	23		After HSCT	NR
1307	54/m	n.d.	34, 33, 13, 65, 15, 19	48		After HSCT	NR
1369	27/m	AML M0	34, 117, 33	70		After HSCT	NR
1375	47/f	AML M5	34, 15, 33, 117	70		After HSCT	NR
1387	73/m	AML M4	117, 33, 13	85		After HSCT	n.d.

AML, acute myeloid leukemia; MDS, myelodysplastic syndrome; CD, Cluster of differentiation; ELN-Risk, European LeukemiaNet Classification 2022; f, female; m, male; FAB, French–American–British classification; IC-Blasts, immune cytologically detected blasts; n.d., no data; NR, no response to induction therapy; R, response to induction therapy; HSCT, relapse after hematopoietic stem cell transplantation.

**Table 3:** Evaluation of cellular composition and cell subtypes by flow cytometry.

Subtypes of DC/DC_leu_				
Name of Subgroup	Referred to	Surface Marker	Abbreviation	Reference
Leukemic blasts	WB/PBMNC	Bla^+^ (CD34, CD117, CD65, CD15)	Bla^+^/WB Bla^+^/PBMNC	[29]
Dendritic cells	WB/PBMNC	DC^+^ (CD1b, CD40, CD80, CD83, CD86, CD206)	DC^+^/WB DC^+^/PBMNC	[29]
Leukemia-derived DC	WB/PBMNC	DC^+^Bla^+^	DC_leu_/WB DC_leu_/PBMNC	[29]
DC_leu_ in DC fraction	DC^+^	DC^+^Bla^+^	DC_leu_/DC^+^	[29]
DC_leu_ in leukemic blast fraction (converted DC_leu_)	Bla^+^	DC^+^Bla^+^	DC_leu_/Bla^+^	[29]
Mature DC	DC^+^	DC^+^CD197^+^	DC_mat_/DC^+^	[29]
Viable DC after cultus	DC^+^	7AAD^−^DC^+^	DC_via_/DC^+^	[29]
Proliferating leukemic blasts	Bla^+^	Bla^+^DC^−^CD71^+^	Bla_prol-CD71_/Bla	[33]
Proliferating leukemic blasts	Bla^+^	Bla^+^DC^−^ipo38^+^	Bla_prol-ipo38_/Bla	[33]
**Subtypes of immune reactive cells for MLC**				
CD3^+^ pan-T cells	WB	CD3^+^	T cells	[34]
CD4^+^ T cells	CD3^+^	CD4+CD3^+^	CD4^+^ T cells	[34]
CD8^+^ Tells	CD3^+^	CD8^+^CD3^+^	CD8^+^ T cells	[34]
Early proliferating T cells	CD3^+^	CD69^+^CD3^+^	T_prol-early_	[34]
Late proliferating T cells	CD3^+^	CD71^+^CD3^+^	T_prol-late_	[34]

PBMNCs, peripheral blood mononuclear cells; WB, whole blood; CD, cluster of differentiation; DC, dendritic cells; DC_leu_, dendritic cells of leukemic origin; Bla, leukemic blasts; MLC, mixed lymphocyte culture.

## Data Availability

The data published in this study are openly available in a public repository with a permanent identifier, such as a DOI.

## Supplementary Materials

The following supporting information can be downloaded at: https://www.mdpi.com/article/10.3390/cancers18050847/s1, Figure S1: DC/DC_leu_ can be generated with standard DC/DC_leu_-generating protocols from blast containing PBMNCs (A1) and with Kits from blast containing WB (A2). Increased concentrations of IL-10, IL-17 and MCP-1 found in DC/DC_leu_-cultures supernatants (B1,C1,D1) as well as MLC supernatants (B2,C2,D2) after incubation with Kits compared to control. Figure S2: Achieved and improved anti-leukemic activity of immunoreactive cells after MLC with PBMNC pre-treated with standard-DC/DC_leu_-protocols or Kits as measured by Cytotoxicity Assay.
