# Supplementary material for: Modulation of Leukemic Blasts into Dendritic Cells (DCleu) and Their Role in Predicting Survival in Patients with AML and MDS
Source: Cancers (Basel). 2026 Mar 6;18(5):847. doi: 10.3390/cancers18050847 (PMC12984686; doi:10.3390/cancers18050847)
Supplement: Supplementary file 1 [file cancers-18-00847-s001.zip › cancers-4131913-supplementary.pdf]

Supplementary Figures:

suppl Figure S1: DC/DCleu can be generated with standard DC/DCleu-generating protocols from blast containing PBMNCs (A1) and with Kits from blast containing WB (A2). Increased concentrations of IL-10, IL-17 and MCP-1 found in DC/DCleu-cultures supernatants (B1, C1, D1) as well as MLC supernatants (B2, C2, D2) after incubation with Kits compared to control.

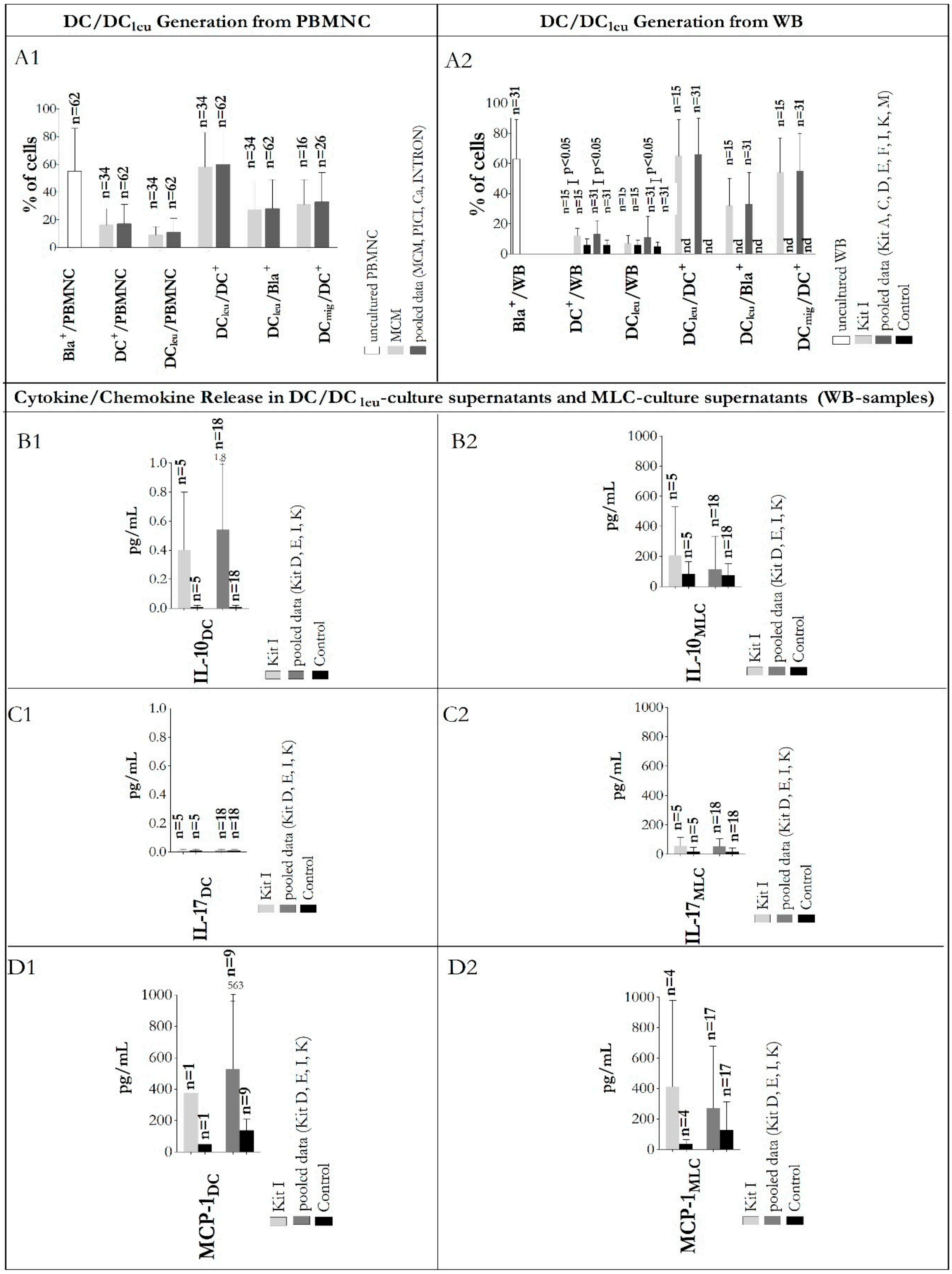

Suppl Figure S1: (A1) Mean ± SD [%] of DC/DCleu subtypes generated from AML-blast-containing PBMNCs using standard protocols (MCM, Ca, PICI, INTRON; pooled data, PBMNC^{DC}) compared to

MCM alone (PBMNC$^{DC(MCM)}$) and uncultured blasts (BLA). (A2) Mean ± SD [%] of DC/DC$_{leu}$ subtypes generated from AML-blast-containing WB with Kits A, C, D, E, F, I, K, and M (pooled data, WB^{DC}) compared to Kit I alone (WB$^{DC(I)}$) and WB without added Kits (control). (B1–D1) Mean ± SD of IL-10, IL-17, and MCP-1 [pg/ml] in DC/DC$_{leu}$-culture supernatants after pre-treatment of WB with pooled Kits (WB^{DC}) compared to Kit I (WB$^{DC(I)}$) and control without added Kits. (B2–D2) Corresponding cytokine/chemokine levels in MLC-culture supernatants. Paired t-test: * p = 0.05–0.1, ** p = 0.005–0.05, *** p < 0.005."

suppl Figure S2: Achieved and improved anti-leukemic activity of immunoreactive cells after MLC with PBMNC pre-treated with standard-DC/DCleu-protocols or Kits as measured by Cytotoxicity Assay.

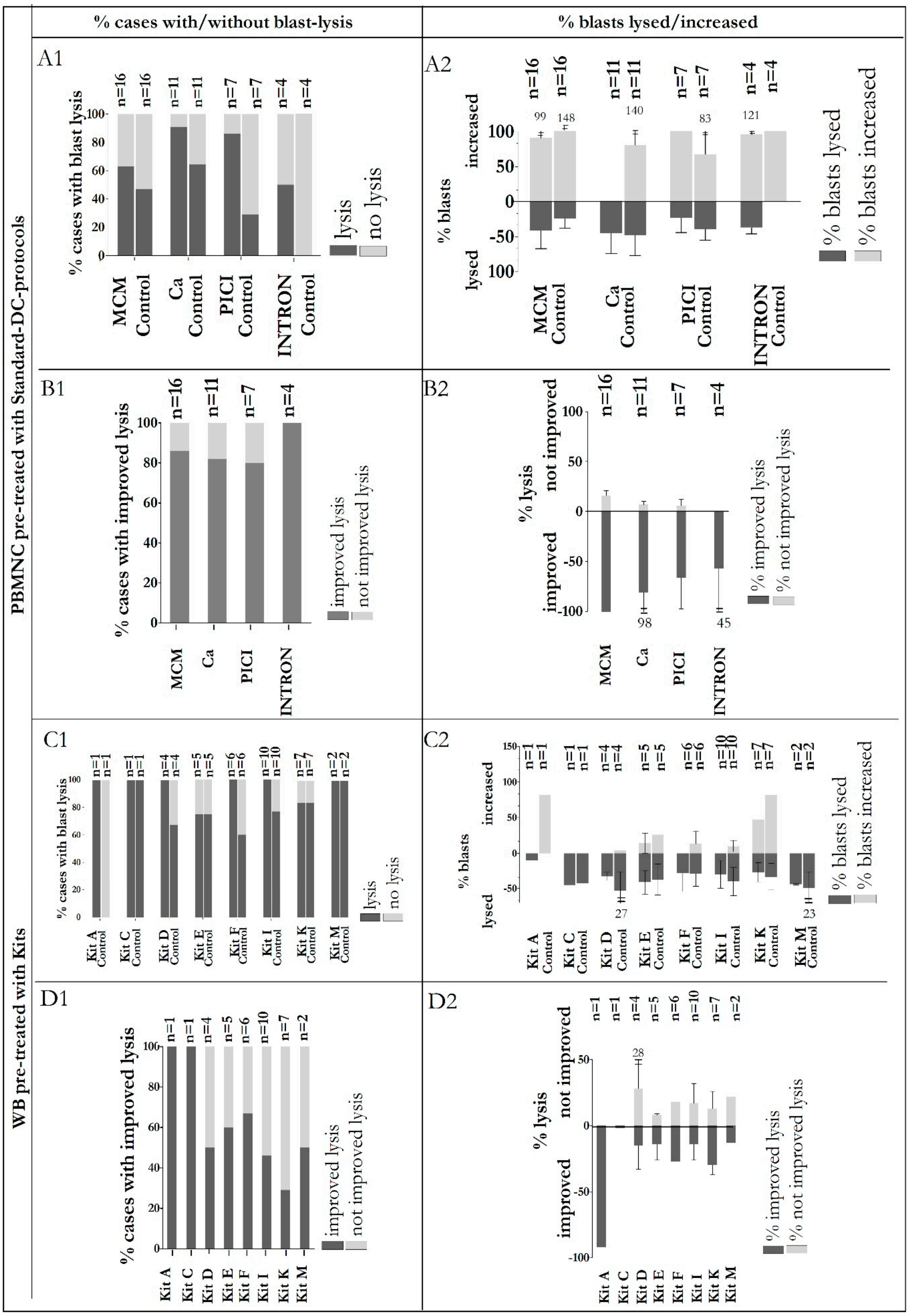

Suppl Figure S2: Lysis of leukemic blasts (anti-leukemic effect) after T cell-enriched MLC. (A1/A2) Cases showing blast lysis after cytotoxic fluorosis assays using T cells stimulated with DC/DCleu generated from leukemic PBMNCs with standard DC/DCleu-generating protocols. (B1/B2) Corresponding improvement of blast lysis compared to control. (C1/C2) Cases showing blast lysis after cytotoxic fluorosis assays using T cells stimulated with DC/DCleu generated from leukemic WB with Kits. (D1/D2) Corresponding improvement of blast lysis compared to control.
